# Tracing the geopolitical influences on the morphological and functional transformation in Guangdong merchant ships: Knowledge mining from the Ming and Qing maritime archives

**DOI:** 10.1371/journal.pone.0336349

**Published:** 2026-01-09

**Authors:** Jinghui Ao, Miao Zhao, Weicong Li, Shengying Feng, Ziying Ye, Zilin Xu, Shanshan Ji

**Affiliations:** 1 School of Art and Design, Guangdong University of Finance and Economics, Guangzhou, China; 2 Design and Creative College of Xiamen University Tan Kah Kee College, Xiamen University Tan Kah Kee College, Zhangzhou, China; 3 Faculty of Built Environment and Surveying, Universiti Teknologi Malaysia, Johor Bahru, Johor, Malaysia; 4 School of Digital Media and Interaction Design, Guangzhou Maritime University, Guangzhou, China; 5 School of Creative Design, Dongguan City University, Dongguan, China; 6 School of Digital Creative Design, Guangdong Nanhua Vocational College of Industry and Commerce, Guangzhou, China; Xiamen University Malaysia, MALAYSIA

## Abstract

Although the institutional history of ancient Chinese maritime trade has been extensively documented, the functional evolution of maritime vessels and their underlying drivers remains underexplored. Recent studies have moved beyond political explanations to explore the interplay of economic and technological dynamics. Using KH Coder for text mining, this study applies word frequency analysis and co-occurrence network modeling to investigate the geopolitical factors shaping the morphological evolution of Guangdong merchant ships in the Ming and Qing dynasties. A visual-comparative analysis further assesses the functional attributes of three representative ship types. Findings reveal that economic and military imperatives were the primary determinants of ship design, with political and geographic factors exerting secondary but supportive influence. For instance, increased piracy threats in the South China Sea prompted structural reinforcements for defensive purposes, while policy shifts under the Canton System encouraged hull designs optimized for high-capacity, long-distance trade. Guangdong’s maritime development was shaped largely by its strategic location and shipbuilding technologies. Ming-era vessels, constructed from teak and cedar, featured brightly painted, flat-bottomed hulls with elevated, streamlined prows. Qing-era ships employed lightweight alloys, muted color schemes, and reinforced double-planked hulls to enhance seaworthiness, while bow structures evolved into sharper and more angular forms. As Guangdong’s maritime trade transitioned from coastal routes to long-distance transoceanic networks—particularly with Europe—its ship design shifted progressively from broad and bulky to agile and eventually more durable configurations. These morphological transformations reflected not only external pressures, such as maritime security concerns and trade expansion, but also internal drivers, including institutional reforms and policy realignments that significantly influenced vessel design. This study contributes to the technical dimension of maritime historiography by emphasizing the merchant ship as an analytical nexus of institutional logic, technological systems, and geopolitical conditions. It offers both theoretical insight and methodological innovation for understanding the mechanisms behind ship design evolution and the spatial organization of premodern Chinese maritime networks.

## 1 Introduction

Ships have been essential tools for the development of human civilization, and their design characteristics and evolution serve as critical indicators of the technological, economic, and socio-cultural conditions of various historical periods. The morphological evolution of merchant ships reflects the relationship between maritime trade and geopolitics, underscoring its relevance to the study of “cross-cultural exchange.” The 1972 Convention Concerning the Protection of the World Cultural and Natural Heritage, adopted by UNESCO, emphasized the necessity of safeguarding maritime cultural heritage [1]. Similarly, Japan implemented the Cultural Properties Protection Law, emphasizing global collaborative actions to safeguard maritime cultural heritage, aiming to preserve cultural diversity and historical authenticity [2]. China’s government also acknowledged the significance of maritime cultural heritage in its 2016 document, Environmental Law Protection of the “Maritime Cultural Routes” Heritage in China, but implementation challenges persist due to limited local enforcement capacity by local authorities [3].

**Fig 1:**
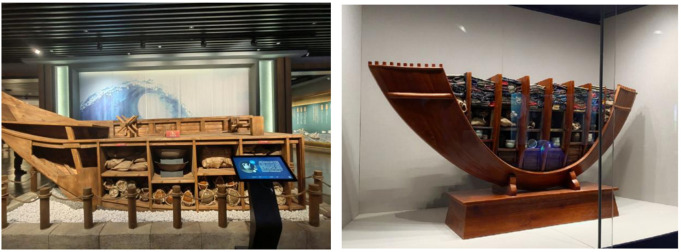
The model of Guangdong merchant ship (Photographed by the author in Guangzhou Maritime Museum).

As a vital carrier of early modern Chinese maritime culture, Guangdong merchant ships gradually developed a regionally distinctive design system and trade-functional structure during the Ming and Qing dynasties (Fig 1). The morphological evolution of these vessels not only mirrored the expansion logic of regional maritime trade networks but also embodied the technological responses and strategic adaptations of southern coastal China to the emerging global maritime order. Within the context of nascent Sino-European maritime corridors and the tribute–commerce hybrid system linking Ryukyu and Japan, Guangdong merchant ships displayed notable diversity and adaptability in structural forms, propulsion systems, and hull compartmentalization, thereby revealing distinct geopolitical and technological characteristics. A systematic investigation into Guangdong merchant ships offers critical insights into the formation of China’s early maritime spatial cognition, clarifies the logic of shipbuilding shaped by institutional-technical interactions, and provides empirical foundations for reconstructing regional maritime heritage. More importantly, such research extends the theoretical boundaries of “ship morphology” within cultural geography and the history of technology. It underscores the necessity of historically reinterpreting and culturally revaluing local vessel typologies, thereby engaging with critiques of material centralism and Western standardization within global heritage discourse.

The evolution of merchant ship morphology functions as a lens through which geopolitical dynamics and the intricate interactions among nations and regions can be observed. However, compared to other themes in maritime tangible heritage, such as customs site ruins [4], historical coastal buildings [5], ceramic trade [6], export painting [7,8], metal artifacts, and weaponry, research on merchant ship morphology remains relatively marginalized. Existing studies on ship morphology primarily emphasize the direct influence of geopolitical factors and their interplay with global trade demands.

Sutrisno [9] demonstrated that maritime security policies directly facilitated the transformation of merchant ships from single-purpose trading vessels into multifunctional defensive platforms. Zhang [10] analyzed regional variations in merchant ship design through the lens of “local decentralization.” Blench [11] extended the role of merchant ship design from national strategies to international dynamics, arguing that power balance directly influenced both the military and economic functions of these vessels. Cheng [12] situated the evolution of merchant ship morphology within an “economic-geopolitical composite model,” suggesting that global economic fluctuations had a direct impact on ship design in China. Monje [13] framed the evolution of merchant ship design within changes to East Asia’s maritime security systems, highlighting how regional security dynamics drove technological innovations in shipbuilding. By applying the “new geopolitics” theory to East Asian maritime studies, Zhao [14] observed that with China’s rise as a maritime power, merchant ship design shifted from addressing traditional security needs to accommodating multifunctional modern requirements.

Zhao [14] examined the interaction between Chinese merchant ship designs and Western shipping technologies, demonstrating that European market demands substantially shaped Chinese vessels with respect to speed, tonnage, and navigational stability. Baker et al. [15] emphasized the strategic importance of ship design, suggesting that the complexity of global trade necessitates innovative functional designs beyond merely meeting existing market requirements. For instance, Ruggiero [16] argued that the coloration of merchant ships functioned as a marker of territorial affiliation and influence. Cariolato [17] demonstrated that merchant ship morphological changes exhibited non-linear relationships with trade demand, emphasizing the intricate interactions between geopolitics and trade requirements.

Recent scholarship has increasingly examined how the evolution of merchant ship morphology has been shaped by the combined forces of geopolitics and global trade demand, and how these forces are reflected in the technical and functional features of ship design. What are the primary drivers of merchant ship design in the context of the complex interactions between global trade networks and geopolitical forces? In what ways do the dynamic interactions between geopolitics and trade demand stimulate technological innovation in merchant ship morphology? The limited exploration of these issues underscores the unique value of this study.

Based on knowledge mining and visual analysis, this study investigates the driving forces behind the morphological evolution of Guangdong merchant ships, positing that their forms were shaped by a constellation of geopolitical factors. From a theoretical perspective, the research reinterprets the morphological features of Guangdong vessels during the Ming and Qing dynasties within a geopolitical framework, providing new insights into the interaction between ship design, national security, and trade policy. From a perspective-driven innovation standpoint, the study incorporates global trade networks into the analysis and extends the examination of ship evolution to encompass technological transformation and international trade competition. To facilitate international readers’ comprehension of culturally specific terminology, S1 Appendix A (Chinese Historical Terms) is included as supplementary material.

The structure of this study is as follows. Section 1 defines the research problem and articulates the core theoretical propositions. A systematic review of the literature traces the developmental stages and theoretical trajectories in the study of merchant ship morphology, highlights existing knowledge gaps, and formulates the research objectives and hypotheses. This section also situates the study within the interdisciplinary nexus of maritime technological history, geopolitical analysis, and cultural heritage studies. Section 2 develops a geopolitical theoretical framework that integrates sovereign governance mechanisms, embedded economic structures, cultural reterritorialization, and militarized spatial logics to systematically account for the dynamic relationship between ship morphology and geopolitical configurations. Sections 2.2–2.3 introduce the quantitative tools and semantic mining techniques employed in the study. Section 3.1 applies KH Coder to perform word frequency analysis and semantic encoding on the textual corpus, extracting the top 100 keywords and grouping them into thematic factor clusters to reveal the multidimensional drivers of Guangdong merchant ship design. Section 3.2 constructs a thematic co-occurrence network model to identify the interactive logic among key factors. It also conducts a co-word analysis of geographic entities to reconstruct maritime routes and spatial configurations of Guangdong merchant ships during the Ming and Qing periods. Section 3.3 constructs a tripartite spatio-temporal-semantic relationship model based on dynasties and keywords to trace the adaptive evolution of merchant ship forms, functions, and institutional frameworks, revealing the structural influence of geo-technological forces across different historical periods.

Section 4 offers a comparative analysis of ship types, functions, and structural features across the two dynasties, exploring their respective patterns of institutional adaptation and technological evolution under distinct political and strategic regimes. Sections 5 and 6 synthesize the research findings, respond to the hypotheses and research questions outlined in the introduction, review the study’s main inferences, compare results with existing literature, highlight unresolved issues, and critically reflect on the study’s limitations. Finally, the study proposes directions for future research aimed at deepening scholarly dialogue at the intersection of geopolitics and the history of technology.

## 2 Methodology

### 2.1 Constructing a geopolitical theoretical framework

**Fig 2:**
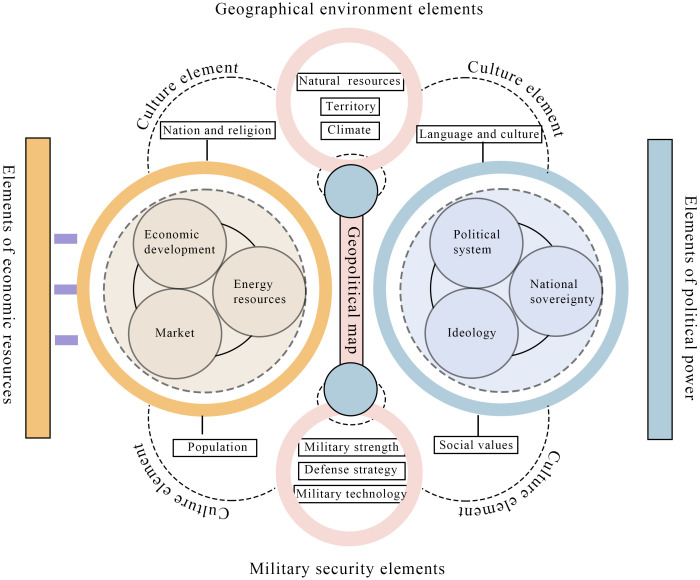
Geopolitical theoretical framework map.

Guangdong merchant ships not only served as vital carriers within the maritime transport and commercial systems of the Qing dynasty but also embodied the material manifestation of geopolitical structures. Their morphological evolution did not follow a single technological trajectory; instead, it resulted from the long-term interplay of power, capital, technology, and culture, demonstrating how geopolitical logics profoundly shape material spatiality. Table 1 illustrates the core concepts and methodology of geopolitical theory, while Fig 2 presents the integrated analytical framework developed in this study. This framework integrates political authority, economic resources, military security, cultural identity, and geographical conditions to clarify how these dimensions interact in reshaping regional spatial configurations. The framework centers on the “Geopolitical Map,” with the left axis emphasizing market mechanisms, energy deployment, and demographic momentum—factors that energize urban spatial dynamics. The right axis underscores the regulatory and integrative effects of state sovereignty, ideology, and governance structures on spatial organization. The vertical axis incorporates natural resources, climate, territorial boundaries, military deployments, and strategic systems. Together, these elements form a holistic mechanism of “structural logic–power operation–spatial differentiation,” with cultural variables mediating the construction of institutional boundaries and spatial belonging.

**Table 1:** The core concepts and research methods of geopolitical theory.

No.	Concepts	Methodologies
1	Power Territorialization emphasizes how state sovereignty, institutional structures, and ideological systems are spatially encoded to construct political order and achieve geographic embedding.	Structure–Function Coupling Analysis is employed to deconstruct the institutional power logic and resource allocation mechanisms underpinning merchant ship morphology. It elucidates how form responds to the compound demands of trade functions, maritime defense strategies, and port network configurations, thereby delineating the interrelated coupling among morphology, function, and authority.
2	Geoeconomic Structuring examines the spatial distribution of economic resources—such as energy, markets, and population—within geopolitical contexts, and how these distributions constrain and shape material-technical systems.	Multivariable Element Integration incorporates five key factors—political authority, economic capital, cultural identity, military deployment, and geographical context—into a unified spatial mapping framework. Through GIS-based modeling and historical data reconstruction, this approach simulates the evolutionary trajectories of ship design under multi-factorial influences, enhancing the interpretive capacity of macro-level analyses.
3	Cultural Re-territorialization highlights how language, religion, and social values contribute to the cognitive construction of spatial affiliation and the delineation of institutional boundaries.	The Institution–Material Translation Model explains how institutional regulations are materialized through choices in construction materials, structural configurations, and manufacturing techniques. This model highlights a dynamic chain linking institutional discourse, technical directives, and material manifestation, revealing how policy texts are operationalized within shipbuilding practices.
4	Militarized Spatial Response explores how military deployments, defense technologies, and strategic frameworks influence the configuration of urban security zones and the structural layout of port facilities.	Cross-Scalar Interaction Mapping analyzes the feedback mechanisms between local, national, and global geotechnical scales. By examining the interactions among institutional expansion, capital circulation, and maritime technological diffusion, this method constructs a dynamic spatial adaptation pathway and technological transmission network, revealing how Guangdong merchant ships achieved geotechnical co-adaptation under multiscalar tensions.

The morphological evolution of ships should be situated within a complex geopolitical framework that accounts for the multidirectional dynamics among geographic topology, technological resource allocation, institutional regulation, cultural semiotics, and military feedback mechanisms. Spatial attributes fundamentally determine the technological adaptability of ship types. For example, inland grain transport favored flat-bottomed hulls, whereas maritime navigation required keeled structures, illustrating technological differentiation shaped by distinct geopolitical regimes. Economic resources delineate the boundaries and trajectories of technological innovation; coastal regions such as Fujian and Guangdong, driven by market forces, fostered ship design optimization, forming a nested model of “capital–technology interdependence.” State governance, through institutional intervention, directed the standardization of state-sponsored shipbuilding, exemplifying a regulatory logic of “technological discipline and vertical integration of power.” Ethnic and cultural identities were embedded in ship morphology through technical aesthetics and functional symbolism. For example, the watertight bulkhead of the Fuchuan was not only a utilitarian feature but also a material expression of clan-based cultural values. Military imperatives served as key drivers of technological iteration; the evolution of warships visibly embodied the state’s capacity for maritime territorial control. The structural transformation of Guangdong merchant vessels illustrates the multidimensional encoding of geopolitical logic in material space. Their evolution reflects not only functional optimization in maritime transport technologies but also the deeper embedding of national institutions, resource governance, and cultural power structures, revealing the theoretical tension and synergistic interaction among morphology, geopolitics, and authority.

### 2.2 Knowledge data mining and research steps

**Fig 3:**
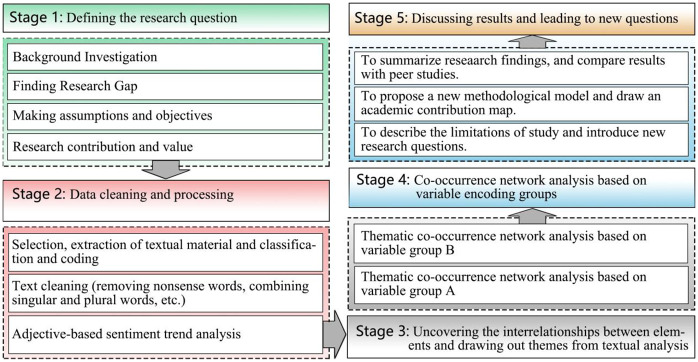
The logic of knowledge data mining.

Knowledge mining is the process of extracting information from large volumes of unstructured text through techniques such as natural language processing and statistical analysis. This study outlines the logical framework of knowledge mining (Fig 3). Stage 1 defines the research questions. Stage 2 entails extracting, categorizing, and encoding textual materials, while identifying factors influencing ship morphology through term frequency (TF) analysis. Stage 3 employs co-occurrence networks generated from the encoded groups to reveal potential relationships between clusters and their components. Stage 4 conducts case studies on the morphology of merchant ships during the Ming and Qing Dynasties, comparing the evolution of Guangdong merchant ship designs across different periods and analyzing the geopolitical factors driving these changes. Stage 5 presents the findings and proposes future research directions.

### 2.3 Analytical tools, data sources and processing

This study employs KH Coder, an open-source software for quantitative text analysis, which offers functions such as word frequency statistics, cross-tabulation, co-occurrence network construction, and multivariate analysis. It supports multiple languages—including Chinese, English, Japanese, and Korean—and integrates functionalities such as text segmentation, term unification, and knowledge graph generation within an intuitive user interface [18].

**Fig 4:**
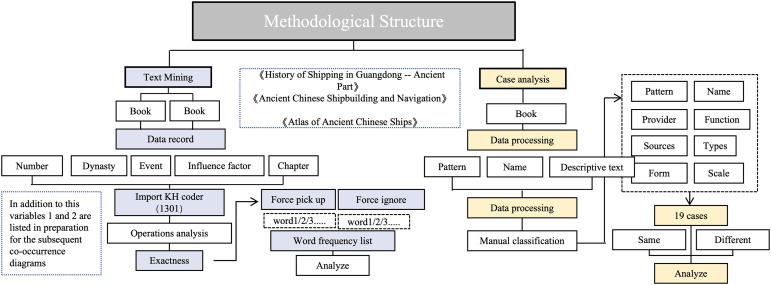
Flowchart of data processing for knowledge mining.

The research integrates text mining and visual analysis methodologies. As illustrated in Fig 4, the data processing workflow encompasses both textual and visual sources. The textual data (S2 Appendix B) used in this study were primarily drawn from History of Guangdong Shipping: Ancient Part by Ye [19] and Ancient Chinese Shipbuilding and Sailing by Zhao [14]. The ship type samples were sourced from a dataset shared on the Figshare platform, comprising 174 entries. These data were extracted from Sailing to Taiwan: A Maritime Chronicle of Taiwanese Boats and Vessels by Lu [20] and Atlas of Ancient Chinese Ships by Wang [21]. Additionally, relevant ship-related information was systematically extracted from classical texts and organized into tabular form (S3 Appendix C). Semantic encoding, keyword extraction, and thematic categorization were performed using KH Coder, forming the basis for variable construction and analytical units. To ensure coding reliability and validity, the research team conducted multiple rounds of collaborative coding. To maintain a focused scope, only merchant ship–related content was extracted, while images, charts, appendices, and non-analytical annotations were excluded. The processed texts were subsequently analyzed for word frequency and operational patterns using KH Coder. To enhance analytical precision, customized “force pick-up” and “force ignore” term lists were applied to suppress semantic noise and emphasize topic-specific keywords [22]. The automatically generated Top Term Frequency List was semantically clustered and employed in co-occurrence network modeling to identify key factors shaping the morphological evolution of Guangdong merchant ships. In section 4 (case study), this study selects 19 representative samples from a total of 174 cases for in-depth analysis (S4 Appendix D). By comparing the visual morphology, structural attributes, and design logics of various ship types, the research elucidates their differentiated trajectories in institutional adaptation, technological evolution, and geopolitical response. This enables an integrated triadic analysis across textual evidence, visual representations, and institutional frameworks.

## 3 Results

### 3.1 Statistics of term frequency (TF)

The data was preprocessed by importing Excel files into KH Coder with the Stanford POS Tagger, followed by verification of the merged terms in the frequency list. The initial statistics revealed 61,626 tokens in the imported documents, of which 35,706 were analyzed. The number of term classes was initially 12,738 but was reduced to 11,686 after term merging. Irrelevant terms such as auxiliary verbs, pronouns, and conjunctions were manually added to the “Force Ignore” list, while semantic refinement and the selection of key terms for the “Force Pick up” list were synchronized (Table 2). A subsequent run excluded 8,247 irrelevant terms and consolidated 258 similar term classes. Using detailed sentence segmentation, the number of analyzable units—including sentences, paragraphs, and cells—was standardized to 1,300. Based on the term frequency descending order panel, a comprehensive list of the top 100 terms (Table 3), a list of the top 30 place names, and a list of the top 20 tags (Table 4) were constructed. S5 Appendix E shows the data on word frequency statistics.

**Table 2:** The list of settings for force ignore, force pick up, and TAG.

Types	Words
Force pick up:	船只; 造船; 造船业; 贸易; 海关技术; 季风; 风向; 地势; 江河; 内河; 远洋; 僧人; 记载;; 货物; 港口; 海禁; 明清; 巡逻; 海盗; 书籍; 交流; 部队; 军队; 官兵
Force ignore:	也; 人; 不; 县; 又; 就; 国; 大; 海; 都; 者; 到; 可; 地; 则; 米; 已; 水; 使; 多; 省; 但; 皆; 粤; 即; 州; 曾; 往来; 能; 来; 渡; 均; 行; 东; 而; 南; 广; 出; 说; 亦; 如; 便; 要; 最; 至; 还; 通; 会; 达; 可以; 很; 路; 约; 舟; 已经; 西; 谷; 银; 仍; 共; 凡; 其中; 郡; 处; 属; 运; 并; 开; 潮; 闽; 糖; 为; 更; 正; 小; 清; 石; 时期; 年间; 发展; 载; 称; 发展; 我国; 出现; 因此; 一带; 赴; 成为; 再; 指出; 事; 入; 北; 主要; 对外; 才; 澳; 买; 余; 作; 业; 江; 河; 只; 曰; 港; 然后; 铅; 下; 于是; 客; 市; 文; 禁; 造; 巡; 帆; 新; 分; 区; 占; 卷; 发; 民; 府; 重要; 而且; 达到; 高; 一; 官; 得; 循; 必须; 月; 此外; 物; 用; 前; 包括; 开始; 故; 设; 先; 卖; 家; 当地; 多以; 指; 条件; 给; 产; 仅; 元; 出海; 却; 口; 形成; 长; 中; 军; 史; 屈; 夷; 常; 收; 数; 派; 甚至; 溪; 到达; 较; 所以; 连
TAG	造船; 记载; 货物; 技术; 船舶; 海禁; 内河; 港口; 交流; 远洋; 僧人; 海盗; 季风; 巡逻; 江河; 地势; 明清; 书籍; 军队; 文人

**Table 3:** Comprehensive word frequency tables for Top 100 words.

No	Words	TF/No.	Weight	No.	Words	TF/No.	Weight
1	Ship (船)	370	100	51	Flotillas (船队)	20	5.40
2	Trade (贸易)	230	62.16	52	Cargo Ship (货船)	20	5.40
3	Shipyard (造船)	106	28.64	53	Import (进口)	20	5.40
4	Coast (沿海)	96	25.94	54	Subdue (镇压)	20	5.40
5	Boats (船只)	95	25.67	55	Factory (厂房)	19	5.13
6	Cargoes (货物)	85	22.97	56	Shibosi (市舶司)	19	5.13
7	Nautical (航海)	84	22.70	57	Navy (海军)	19	5.13
8	Overseas (海外)	78	21.08	58	River Way (河道)	19	5.13
9	Maritime (海上)	77	20.81	59	Book (书籍)	18	4.86
10	Merchant Ship (商船)	76	20.54	60	Goods (商品)	18	4.86
11	Qing Dynasty (清代)	72	19.45	61	Large Ship (大船)	15	4.05
12	Businessman (商人)	66	17.83	62	Paddle (船桨)	15	4.05
13	Quantity of goods (货量)	63	17.02	63	Sea Freight (海运)	15	4.05
14	Salt (盐)	56	15.13	64	Foodstuff (粮食)	15	4.05
15	Skill (技能)	50	13.51	65	Coastal Defence (海防)	15	4.05
16	Boatman (船户)	44	11.89	66	Island (岛屿)	15	4.05
17	Qing Qianlong (清乾隆)	39	10.54	67	Transhipment (转运)	15	4.05
18	Government (政府)	39	10.54	68	Silk (丝绸)	14	3.78
19	Transport (交通)	37	10	69	Peddle (小贩)	14	3.78
20	Area (地区)	37	10	70	Provincial Guild Hall (省级会馆)	14	3.78
21	Haulage (运输)	31	8.37	71	Status (地位)	14	3.78
22	River (内河)	31	8.37	72	City Wall (城墙)	14	3.78
23	Authorities (当局)	31	8.37	73	Wood (木头)	14	3.78
24	Sailboat (帆船)	31	8.37	74	Sailor (水手)	14	3.78
25	Transport by Water (水运)	29	7.83	75	Sandboat (沙船)	14	3.78
26	Custom (海关)	28	7.56	76	Archaic (古老)	14	3.78
27	Port (港口)	28	7.56	77	Product (产品)	14	3.78
28	Route (路线)	27	7.29	78	Treasure Ship (宝船)	14	3.78
29	Scale (尺度)	26	7.02	79	Structure (结构)	14	3.78
30	Merchant (商贾)	26	7.02	80	Pirates (海盗)	14	3.78
31	Warship (战船)	25	6.75	81	Conduct Commerce (通商)	13	3.51
32	Economy (经济)	25	6.75	82	Copper (铜)	13	3.51
33	Vessel (船艇)	25	6.75	83	Inhabitant (居民)	13	3.51
34	Turreted Junk (楼船)	25	6.75	84	Isles (岛屿)	13	3.51
35	Naval Blockade (海禁)	25	6.75	85	Warfare (战争)	13	3.51
36	Ocean Liner (海船)	24	6.48	86	Buddha (佛)	13	3.51
37	Coin (钱币)	24	6.48	87	History (历史)	13	3.51
38	Watercraft (水上载具)	23	6.21	88	Bow (船头)	13	3.51
39	Navigate (航行)	22	5.94	89	Viceroy (总督)	13	3.51
40	State (州属)	21	5.67	90	Quicksand (河沙)	13	3.51
41	Formulation (制定)	20	5.40	91	Yamen (衙门)	13	3.51
42	Ferry (渡船)	20	5.40	92	Wind Speed (风速)	13	3.51
43	Toll (通行费)	20	5.40	93	Passage (航程)	12	3.24
44	Rule (规则)	20	5.40	94	Sea-going Vessels (海船)	12	3.24
45	Qing Kangxi (清康熙)	20	5.40	95	Cropland (田地)	12	3.24
46	Ship Type (船型)	20	5.40	96	Outlet (出口)	12	3.24
47	Jetty (码头)	20	5.40	97	Fish (鱼)	12	3.24
48	Culture (文化)	20	5.40	98	Dealings (交易)	11	2.97
49	Seashorest (海滨)	20	5.40	99	Official Residence (官邸)	11	2.97
50	Hull (船体)	20	5.40	100	County Annals (县志)	11	2.97

**Table 4:** A list of place words and TAG words for Top 30 words.

Locations				TAG			
No	Words	TF/No.	Weight	No.	Words	TF/No.	Weight
1	Guangdong (广东)	238	100	1	Shipyard (船坞)	106	100
2	Guangzhou (广州)	197	82.77	2	Record (记录)	95	89.62
3	China (中国)	136	57.14	3	Cargoes (货物)	85	80.18
4	Foshan (佛山)	79	33.19	4	Skill (技能)	50	47.16
5	Nanhai Sea (南海)	64	26.89	5	Watercraft (水上载具)	35	33.01
6	Chaozhou (潮州)	64	26.89	6	Naval Blockade (海禁)	34	32.07
7	Fujian (福建)	56	23.52	7	River (河流)	33	31.13
8	Macao (澳门)	43	18.06	8	Seaport (海港)	31	29.24
9	India (印度)	39	16.38	9	Exchange (交换)	9	8.49
10	Japan (日本)	38	15.96	10	Distant seas (远海)	9	8.49
11	Chenghai (澄海)	35	14.70	11	Monks (僧侣)	7	6.6
12	Hainan (海南)	34	14.28	12	Piracies (海盗行为)	7	6.6
13	Pearl River (珠江)	30	12.60	13	Monsoon (季风)	3	2.83
14	Quanzhou (泉州)	25	10.50	14	Patrol (巡逻)	3	2.83
15	Guangxi (广西)	23	9.66	15	Great rivers (大河)	3	2.83
16	Chaoyang (朝阳)	22	9.24	16	Topography (地貌)	2	1.88
17	Panyu (番禺)	22	9.24	17	Ming & Qing Dynasties (明清时期)	2	1.88
18	Jiangsu (江苏)	20	8.40	18	Work (工作)	1	0.94
19	Jiangxi (江西)	20	8.40	19	Service (服务)	1	0.94
20	Shanghai (上海)	19	7.98	20	Laborer (劳工)	1	0.94
21	Jiangmen (江门)	19	7.98	21			
22	Qiongzhou (琼州)	19	7.98	22			
23	Shunde (顺德)	19	7.98	23			
24	Dongguan (东莞)	18	7.56	24			
25	Taiwan (台湾)	18	7.56	25			
26	Shandong (山东)	16	6.72	26			
27	Haikou (海口)	15	6.30	27			
28	Britain (不列颠)	14	5.88	28			
29	Leizhou (雷州)	14	5.88	29			
30	Gaozhou (高州)	14	5.88	30			

To normalize the data and minimize potential bias, term frequencies were weighted using Equation 1. Zhang et al. [23] applied this normalization formula to datasets with different linguistic structures and verified its stability in lexical clustering analysis. The method effectively reduces biases caused by differences in word frequency while preserving meaningful distinctions among terms. The equation was developed to standardize term frequency data across different semantic categories, thereby minimizing distortions caused by discrepancies in raw frequency values. This approach parallels the principle of TF-IDF, wherein each term frequency is normalized by dividing it by the maximum observed frequency ${\text{N}}_{\text{max}}$ within the dataset, yielding a standardized value between 0 and 100. This normalization enhances the consistency and comparability of terms across semantic groups [24]. In this study, as presented in Table 5, the vocabulary has been classified into six semantic categories, with total frequencies and weighted scores calculated for each group to improve the interpretability and statistical robustness of the analysis. Additionally, during the preprocessing stage, multiple rounds of term merging and semantic refinement (section 3.1) were conducted. This iterative process also served as an implicit sensitivity analysis to ensure the stability of term classification and the methodological reliability of the frequency analysis.

**Table 5:** Semantic classification of words in Table 3.

Groups	Name	Words	TF/No.	Weight
A	Economy	Trade, Cargoes, Businessman, Quantity of Goods, Salt, Boatman, Coin, Haulage, Transport by Water, Custom, Seaport, Merchant, Economy, Toll, Import, Factory, City Hublot, Sea Freight, Product, Conduct Commerce, Copper, Transhipment, Silks, Peddle, Outlet, Caviar, Dealing, Foodstuff, Navigate	983	265.53
B	Vehicle	Navy, Warfare, Coastal Defence, Sailor, Japanese Pirate	977	263.95
C	Others	Nautical, Overseas Country, Qing Dynasty, Skill, Qianlong, Area, State, Scale, Kangxi, Culture, Subdue, Book, Structure, Inhabitant, Status, City Wall, Wood, Buddha, History, Cropland, Official Residence, County Annals	614	165.86
D	Geography	Coast, Maritime, Transport, River, Route, Ferry, Jetty, River Way, Seashorest, Isle, Island, Quicksand, Wind Speed, Passage	413	111.56
E	Politics	Governments, Naval Blockade, Authority, Formulation, Rule, Provincial Guild Hall, Viceroy, Yamen	155	41.86
F	Military	Ship, Shipyard, Boat, Merchant Ship, Watercraft, Sailboat, Vessel, Ocean Liner, Hull, Flotillas, Cargo Ship, Large Ship, Paddle, Archaic, Sandboat, Warship, Treasure Ship, Turreted Junk, Bow, Sea-going Vessel, Ship Type	75	20.25


$$N_{a}^{\prime }=N_{a}\cdot \text{100}/N_{\text{max}}$$ (1)

In the equation, a represents a specific term; $N_{a}$ denotes the frequency of the term a; $N_{\text{max}}$ indicates the maximum value of the $N_{a}$ of all term frequencies in the dataset; $N_{a}^{\prime }$ represents the converted weight value.

### 3.2 Coding theme-based co-occurrence network and location co-word relevance analysis

**Fig 5:**
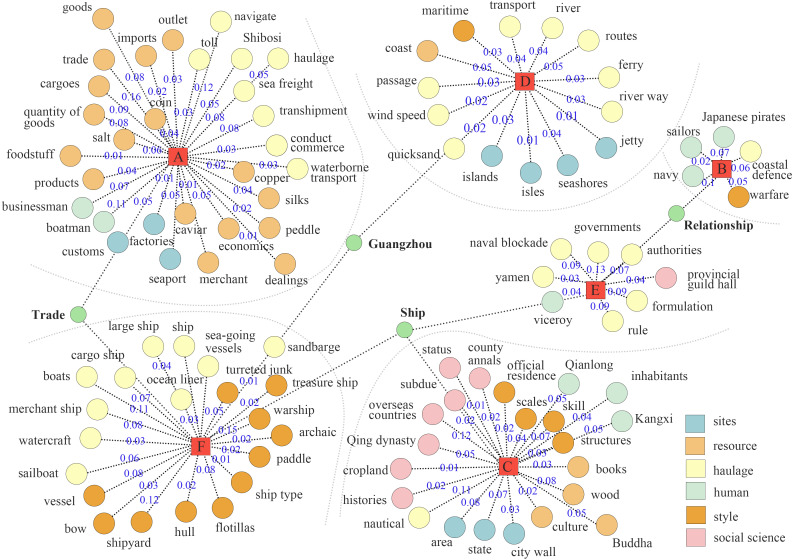
Co-occurrence networks based on lexical groupings in Table 5.


Using the categorized terms from Table 5, a thematic network comprising “nodes” (terms) and “edges” (connections) was constructed for the top 100 terms. The co-occurrence network was visualized as a minimum spanning tree (Fig 5), representing the fundamental relationships between terms with the least number of connecting edges. This approach allows researchers to identify thematic clusters within the text. Co-word analysis, as applied here, uncovers latent associations between terms by identifying words that frequently co-occur within the same text. This study calculates the correlations between words using Equation 2, with the Jaccard coefficient adopted as the computational rule [25]. The Reading Value (RV) at each node is generated based on textual semantics, and the software clusters words into groups of different colors according to their RV and textual associations. A higher RV indicates that the corresponding term holds greater significance within its cluster.

Jaccard (A,B)=|A∩B|/|A∪B| (2)

In this equation, A and B denote two lexical items under calculation. The Jaccard coefficient is obtained by dividing the intersection of A and B by their union, thereby generating the co-occurrence network of textual data. This coefficient measures the frequency with which words A and B appear together within the same textual unit, thus revealing the strength of their association [25].

In Cluster A (economy), high-RV terms include goods (RV = 0.08), imports (RV = 0.02), trade (RV = 0.16), salt (RV = 0.06), seaport (RV = 0.05), merchant (RV = 0.01), and factories (RV = 0.01). This indicates that economic functions and commodity circulation were the primary drivers in the formation and development of Guangdong’s maritime trade networks. It can therefore be inferred that the early evolution of merchant ship morphology was largely determined by exchange efficiency and logistical demands, rather than by purely military or political considerations. In Cluster D (geography), the concentration of terms such as maritime (RV = 0.03), coast (RV = 0.05), ferry (RV = 0.03), jetty (RV = 0.01), and seashores (RV = 0.04), together with wind speed (RV = 0.02), quicksand (RV = 0.02), and river way (RV = 0.03), demonstrates that natural geography and hydrological conditions directly shaped shipping corridors. The evolution of Guangdong shipbuilding technology adapted to these environmental uncertainties, with ecological constraints becoming critical references for hull design and route planning. Cluster C (politics) aggregates terms such as Qing Dynasty (RV = 0.05), county annals (RV = 0.02), official residence (RV = 0.02), and structures (RV = 0.03). These terms reveal that state authority incorporated maritime activities into its governance framework through institutions and archival practices. In Cluster F (military), frequent terms include cargo ship (RV = 0.07), warship (RV = 0.15), turreted junk (RV = 0.05), and flotillas (RV = 0.08). This suggests that Guangdong vessels combined both commercial and defensive functions. In particular, the juxtaposition of merchant ship (RV = 0.08) and warship (RV = 0.15) highlights how piracy, naval blockades, and geopolitical competition drove the hybridization of merchant vessels, reflecting the profound influence of geopolitics on their morphological development.

**Fig 6:**
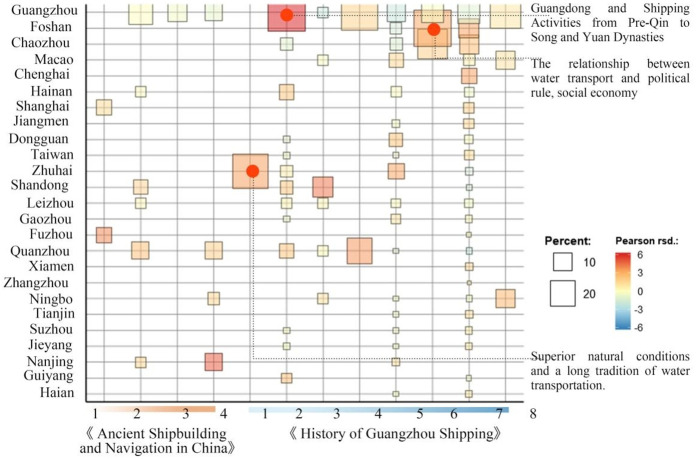
Location word association analysis in different data sources.

Additionally, the top 25 Chinese place names were encoded to create Fig 6, illustrating the co-occurrence results of events mentioned in different texts concerning these place names. Fig 6 presents the co-occurrence relationships between geographic locations and textual sources, represented by rectangular nodes. The size of each rectangle reflects the frequency of a specific location’s appearance within a given source—the larger the rectangle, the more frequently the location is mentioned. Color coding, together with Pearson residuals (rsd.), denotes both the strength and direction of correlations between locations and textual content: red indicates positive correlations, blue negative correlations, and darker shades represent stronger associations. The combined use of percentage values and color gradients illustrates both the frequency and correlation intensity of location mentions. The 10% and 20% thresholds in the legend denote the distribution ranges of such co-occurrence patterns within the dataset.

### 3.3 Co-occurrence correlation analysis based on dynasties and keywords

**Fig 7:**
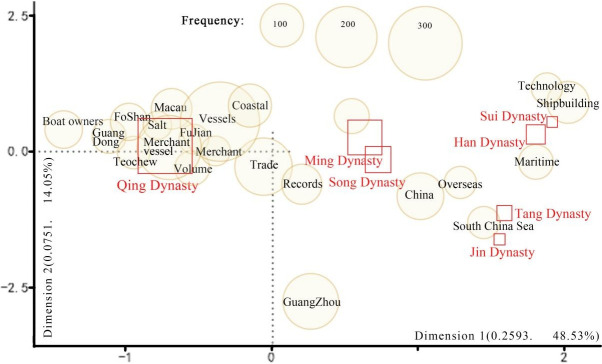
Results of lexical MDS analysis based on different dynasties.

Using the ‘Correspondence Analysis of Words-Select Words’ feature in KH Coder, this study filtered words with a minimum term frequency (Min. TF) of 40 and generated a bubble plot (Fig 7) to depict Multidimensional Scaling (MDS) analysis results across seven dynasties. The X-axis (Dimension 1) and Y-axis (Dimension 2) account for 48.53% and 14.05% of the variance, respectively, forming a reduced two-dimensional spatial configuration as indicated by the dashed lines [26]. Dimension 1 captures the primary characteristics of the original data, explaining 48.53% of its variance; words positioned closer along this axis tend to exhibit higher semantic proximity. In contrast, Dimension 2 explains only 14.05% of the variance, making Dimension 1 more analytically meaningful for interpreting co-occurrence patterns [27]. Collectively, the two dimensions explain 62.58% of the total variance, indicating statistical robustness and a reliable approximation of inter-word relationships [28]. Furthermore, the MDS visualization highlights the semantic salience of terms located near the intersection of the dashed lines—an area of centrality—based on their proximity to this focal point [29]. The size of the circles represents TF, with larger circles indicating higher frequencies. The parameter panel in KH Coder does not provide functions for calculating or displaying the stress value. This limitation will be addressed in future research, where our team plans to conduct more in-depth investigations.

Chi-square analysis is a widely used statistical method for evaluating the association between lexical items and categorical variables such as document types, historical periods, or geographical regions [30]. A higher chi-square value indicates a significant deviation between the observed and expected frequencies of a given term, suggesting that the term appears disproportionately in one category compared to others. This method is commonly applied in data classification, temporal trend analysis, and regional variation studies. Using Equation 3, this study adopts a hybrid calculation approach to assess the correlation between lexical terms and dynastic/geographical categories, as summarized in Table 6. While the magnitude of the chi-square value is positively correlated with the strength of association between variables, it does not imply causality.

**Table 6:** Chi-square analysis based on dynastic and locational words.

	Countries											
Periods	China	India	Japan	UK	LKA	Arab	Vietnam	Siam	SHP	PRK	NLD	PRT
Ming Dynasty	33 (13.36%)	26 (10.53%)	8 (3.24%)	0 (0.00%)	10 (4.05%)	7 (2.83%)	4 (1.62%)	1 (0.40%)	0 (0.00%)	5 (2.02%)	0 (0.00%)	0 (0.00%)
Qing Dynasty	26 (4.32%)	2 (0.33%)	8 (1.33%)	11 (1.83%)	0 (0.00%)	0 (0.00%)	1 (0.17%)	7 (1.16%)	9 (1.50%)	0 (0.00%)	6 (1.00%)	1 (0.17%)


$$\chi^{\text{2}}=\sum \frac{{\left(O_{i}-E_{i}\right)}^{\text{2}}}{E_{i}}$$ (3)

In the equation, $\chi^{\text{2}}$ represents the Chi-square; i denotes a specific term; ${\text{O}}_{\text{i}}$ indicates the actual TF of the word i within a particular category; and $E_{i}$ represents the expected TF of the word i under the null hypothesis of no significant difference, calculated based on the total number of samples and the proportion in each category, assuming no association between the two variables.

## 4 Case study

### 4.1 The analysis of the common ship types and the three main merchant ship forms of Guangdong shipping in the Ming and Qing dynasties

**Fig 8:**
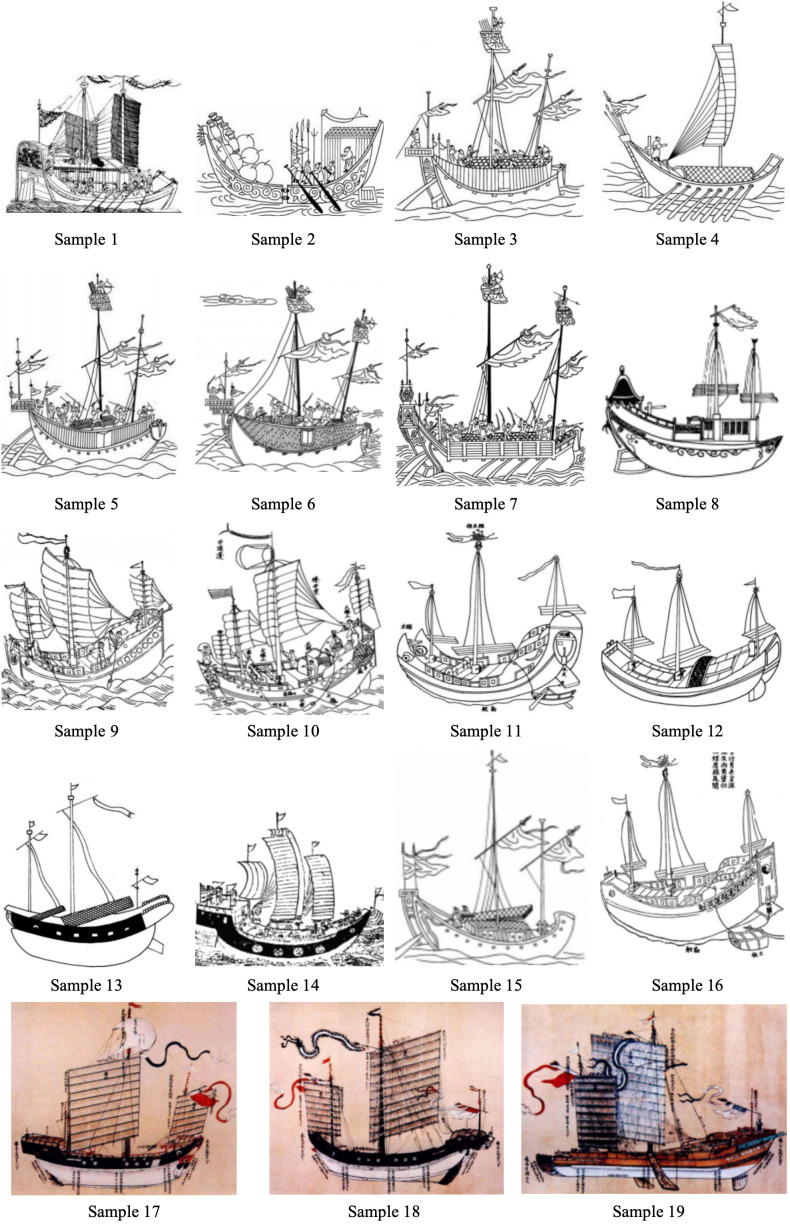
Atlas of Ship Types in Guangdong during the Ming and Qing Dynasties.

Fig 8 presents the coding of 19 Guangdong merchant vessels from the Ming and Qing dynasties. Table 7 summarizes key information about these ship types and facilitates the examination of three core analytical dimensions. First, it assesses whether the structural reconstructions of these vessels in specific historical contexts reflect a dual orientation toward functional optimization and institutional adaptability. Second, it explores whether the morphological evolution of ship types reveals a pattern of technological path dependence intertwined with embedded regional knowledge. Third, it evaluates the extent to which ship types can serve as spatial indicators for identifying shifts in maritime spatial order and coastal development regimes in premodern China. Through this analytical framework, the study seeks to move beyond the conventional dichotomy between textual documentation and material evidence in maritime research. It proposes a tripartite methodological paradigm that integrates visual representation, structural morphology, and institutional context, thereby offering a multidimensional, empirically grounded basis for understanding the evolutionary logic of ancient Chinese maritime technological systems.

**Table 7:** Types of ships in Guangdong.

	Ming Dynasty			
Sample	Sample 1	Sample 2	Sample 3	Sample 4
Name	Caofang Ship (漕舫船)	Chain-linked Ship (联环舟)	Straw-paved Ship (草撇船)	Bugle Ship (叭喇唬船)
Provider	Song Yingxing (宋应星)	Mao Yuanyi (茅元仪)		
Sources	Tiangong Kaiwu (天工开物)	Wubei Zhi (武备志)		
Types	Freight transport	Naval defense		
Function	Inland Waterway Grain Transport	Warship		Chase ship
Form	The bow and stern of the ship are square in shape, with a flat bottom and shallow draft.	The ship’s hull is divided into a front section that constitutes one-third of the total length and a rear section that makes up the remaining two-thirds, connected in the middle by rings.	The ship is equipped with a small boat that is typically hung on one side of the ship’s side. The components such as the bow cap and rudder plate are made of camphor wood.	The ship has a pointed bottom and a wide deck, with identical shapes at both ends. A keel runs through the bottom, extending from the bow to the stern.
Scale	The vessel has a length of 52 feet and a width of 9 feet 5 inches, with a carrying capacity of 2,000 stones of rice.	The boat is approximately 40 feet long and relies on oars for propulsion.	The boat is 75 feet long, with an aft overhang of 10 feet, an interior depth of 8 feet, and planks that are 2.5 inches thick.	The vessel measures 62 feet in length and 5 feet 2 inches in depth, with side supports made of wooden planks 2.3 inches thick.
Sample	Sample 5	Sample 6	Sample 7	Sample 8
Name	Haicang Ship (海沧船)	大Fu Ship (福船)	Guang Ship (广船)	Big Yellow Ship (大黄船)
Provider	Hu Zongxian (胡宗宪)			Li Zhaoxiang (李昭祥)
Sources	Chouhai Tubian (筹海图编)			Longjiang Chuan Chang Zhi (龙江船厂志)
Types	Naval defense		Freight transport, Warship	Royal Passenger Ship
Function	Warship		Maritime transport	
Form	The ship is fitted with one large sail and one small sail, two large oars, two rudders, and three wooden anchors.	The ship is tall, resembling a building, and can accommodate over a hundred people. It features a narrow, sharp bottom with a wide upper section.	This is a pointed-bottom sea vessel with a narrow base and a broad upper section, resembling two wings. It is stable in coastal waters but tends to sway in open seas.	The ship’s bottom is approximately 2.2 inches thick. Both the bow and stern are square and decorated with a stone yellow coating.
Scale	The draft of the boat ranges from 7 to 8 feet.	The boat has a length of 90 feet, with an aft overhang of 13 feet, an interior depth of 13 feet, and planks that are 2.5 inches thick.	The vessel is 100 feet long and over 30 feet wide.	The boat measures 79.3 feet in length, 52.4 feet in depth, and 20.2 feet in width.
	Qing Dynasty			
Sample	Sample 9	Sample 10	Sample 11	Sample 12
Name	Dan Ship (蜑船)	Sha Ship (沙船)	San Bu Xiang Ship (三不像船)	Defensive ship
Provider	–	–	–	–
Sources	Jiangsu Marine Transportation Quanan (江苏海运全案)			
Types	Freight transport			
Function	Ocean Shipping			
Form	The ship’s hull is relatively long with a deep hold, and both the bow and stern are square. A standard-sized ship of this type can carry up to 1,800 stones.	The ship has a flat bow, a square stern, a flat bottom, and a broad body with multiple masts and sails. It has a shallow draft.	The ship has a long body with a wide belly, a pointed bow, and a high stern. The bottom is coated with oyster powder, and the bow and stern are painted with red alum water.	The ship has a long body and a wide belly, with a low bow and stern. The masts are short, and there are no awnings or side decorations.
Scale	The vessel is equipped with three masts and four sails. It is similar to the ‘San Bu Xiang Ship (三不像船)’ but slightly smaller.	The boat has a length of 100 feet, a carrying capacity of 1,500 shi (石), and is equipped with three masts and four sails.	When fully loaded, the vessel carries 2,000 stones and is equipped with three masts and four sails.	The boat is fitted with three masts.
Sample	Sample 13	Sample 14	Sample 15	Sample 16
Name	Ju Ship (锯船)	Fengzhou Official Ship (封舟官船)	Bird Beak Ship (鸟嘴船)	Zhe Ship (蜇船)
Provider	–	Xu Baoguang (徐葆光)	Song Yingxing (宋应星)	–
Sources	Shuishi Jiyao (水师辑要)	Zhongshan Chuaxin Records (中山传信录)	Tiangong Kaiwu (天工开物)	Atlas of Ancient Chinese Ships (中国古船图谱)
Types	Naval defense	Official Ship (官船)	Freight transport, Naval defense	Freight transport
Function	Warship	Passenger ship	Reconnaissance of fishing ship	North Sea Ocean Shipping
Form	It is constructed of pine wood.	The ship has a pointed bow and a high, square stern.	The shape of the bow resembles a bird’s beak.	The ship has a long hull and a deep hold, with both the bow and stern square in shape.
Scale	The vessel is 89 feet long, 22 feet 5 inches wide, 7 feet 9 inches deep, with planks 3.1 inches thick, and a main mast height of 82 feet.	The boat measures 100 feet in length, 28 feet in width, and has a hold depth of 15 feet, with three masts.	The length ranges from 40 to 50 feet.	The ship has a carrying capacity of 1,800 stones and is equipped with three masts and four sails.

The selection of the 19 ship samples (S4 Appendix D) in this study was guided by the following criteria. The first is chronological representativeness. The ship types span the Ming and Qing dynasties, capturing major transformations in state policy, maritime expansion, economic networks, and shipbuilding technologies. This temporal breadth enables the construction of a continuous morphological evolution chain, facilitating analysis of the inland–coastal–oceanic transition mechanisms. The second is functional diversity. The sample includes vessels serving diverse purposes—grain transport, maritime trade, military defense, passenger conveyance, official duties, and reconnaissance—reflecting the complex division of maritime labor and the structural diversity of trading networks. For example, the Caofang Ship (Sample 1) was designed for inland navigation, while the Fu Ship (Sample 6) and Sha Ship (Sample 10) were tailored for dual trade and defense functions. The third is geopolitical spatial adaptability. Ship types were selected for their relevance to the coastal waters of Guangdong and the South China Sea–Southeast Asia maritime corridor. These vessels exhibit regional adaptation in hull design and performance, such as the Guang Ship, Dan Ship, and Fengzhou Official Ship, providing insight into how geographic environments influenced structural configurations. The fourth is morphological distinctiveness.

The selected ships exhibit distinct design characteristics, such as keel configuration, cabin partitioning, sail arrangement, and hull proportions. These traits can be consistently coded across textual and visual sources, enabling standardized comparative analysis and cross-referencing between image and document. The fifth is documentary verifiability. Each vessel type is referenced in authoritative historical texts and illustrated sources, thereby ensuring empirical reliability and archaeological validity. The sixth is institutional typological diversity. The selection reflects variations in institutional affiliation—state-operated vessels (e.g., Fengzhou Official Ship), commercial vessels (e.g., Bird Beak Ship), military ships (e.g., Fu Ship), and hybrid models (e.g., San Bu Xiang Ship)—allowing for analysis of how institutional logic is translated into technological and morphological outcomes. The seventh is cultural semiotic embedding. Priority is given to ships featuring ornamental motifs, naming conventions, and color codes, revealing how cultural identity, religious symbolism, and regional aesthetics were materialized in vessel design. For instance, the “red forehead and bird-eye” motif of the Red-Head Ship provides a representative case for extended analysis. The eighth aspect is dimensional and performance contrasts. The sample includes ships with significant variation in length, beam, draft, and tonnage, enabling the modeling of size–function relationships. The Fu Ship, for example, measures up to 90 feet and accommodates over 100 people, while the Sha Ship features a wide and shallow hull suited to shallow-water commerce.

**Fig 9:**
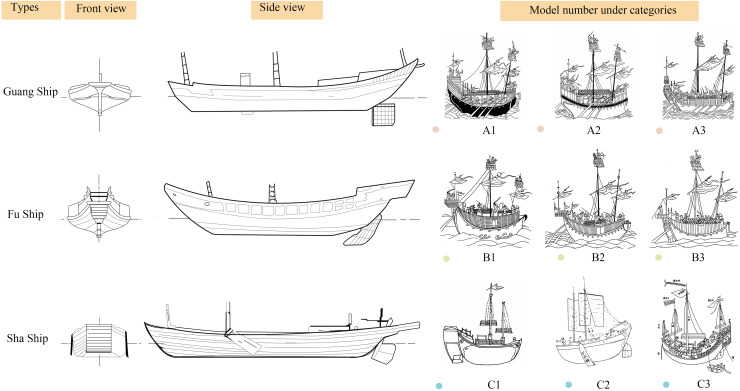
Comparing the Three Major Ship Types of Guangdong Merchant Ships in the Ming and Qing Dynasties.

Fig 9 compares the three primary types of merchant vessels from the Ming and Qing dynasties—Wu Cao Ship (乌艚船), White Cao Ship (白艚船), and Sha Ship (沙船)—while Table 8 documents their corresponding technical and historical attributes. These vessel types exemplify the functional and morphological diversification of commercial shipping during the period and represent distinct evolutionary pathways driven by varying maritime demands. Specifically, groups A1–A3 correspond to Guang Ships, B1–B3 to Fu Ships, and C1–C3 to Sha Ships. Through systematic coding and comparative analysis of structural forms, dimensional proportions, propulsion systems, and constructional details, this study reconstructs their morphological trajectories and investigates the historical factors shaping them. Employing an integrated methodological framework that combines visual historiography with techno-material cultural analysis, the research seeks to elucidate the diachronic logic of ship-type transformation. It further analyzes the structural characteristics shaped by geopolitical shifts, changing navigational functions, technological innovations, and institutional regulations.

**Table 8:** Information about the three major ship types of the Ming and Qing dynasties.

Name	Guang Ship (广船)	Fu Ship (福船)	Sha Ship (沙船)
Sample	Sample 17	Sample 18	Sample 19
Types	It is a large, pointed-bottomed seagoing vessel and comes in many models, such as the Yokogawa, the Pointed Tail, the Big Head, and the Red Head.	It has many aliases, such as grass skimmer, seacraft, whistle boat and winter boat.	It is categorized into four types of body types: extra large, large, medium and small.
Periods	Tang to Qing Dynasty	Song to Qing Dynasty	Tang to Qing Dynasty
Materials	Ironwood	Pine and fir	Camphor wood
Morphology	The boat is huge and has a long pointed head and a wide top and narrow bottom.	The bottom of the ship has a pointed rounded and small square head, while its broad stern with a parapet resembles a platform.	The ship has a flat head, square helm and flat bottom, while the hull is wider and has more masts and sails.
Colors	Pink, Red, White, Black	Green, White, Black, Stone Yellow	Red, White, Blue
Transport	Inland Sea	Inland Sea, Outer sea voyage	Inland and river transportation
Advantages	It has a strong hull to keep out the wind and waves and to set fires.	It resists sinking well and is suitable for long voyages.	It has a shallow bottom draft.
Defectives	It is expensive and difficult to repair. It is more stable in the inner sea, but less stable in the outer sea.	It has poor durability and is not resistant to insects.	It has a large area of water on the bottom and the boat has poor wave breaking ability.

**Noted:** A1 = Pointed Stern Ship; A2 = Blunt Bow Ship; A3 = Guangdong Ship; B1 = Dafu Ship (大福船); B2 = Haicang Ship (海沧船); B3 = Straw-paved Ship (草撇船); C1 = Erbaixun Sha Ship (二百巡沙船); C2 = Sha Ship 1 (沙船1); Sha Ship 2 (沙船2).

### 4.2 Analysis of events related to the evolution of Guangdong ship design

Table 9 provides a systematic overview of key historical events closely related to the morphological evolution of ships in Guangdong. These events are categorized into multiple levels of influence according to their impacts across political, economic, technological, and cultural dimensions, thereby constructing a causal chain linking events, mechanisms, and ship morphology. This multi-source driving structure underlying the evolution of Guangdong ship design reveals a deeply embedded coupling between historical events and material forms.

**Table 9:** The events related to Guangdong ship design.

No.	Periods	Events
1	Ming Dynasty	The Fu Ship was a sharp-bottomed seagoing vessel constructed primarily along the coastal regions of Fujian and Zhejiang during the Ming dynasty. Its design featured a pointed keel, flared hull, elevated bow with an open mouth, raised stern, multiple deck layers, and watertight bulkheads, making it particularly suited for both navigation and combat.
2		Structurally, the Fu Ship comprised four distinct functional levels. The prominently raised bow was often equipped with ramming devices, while its combat platforms supported both archery and cannon deployment, rendering the vessel highly effective in naval warfare.
3		The fleet led by Zheng He represented the apex of premodern Chinese shipbuilding and maritime capabilities. However, its development was ultimately curtailed by the implementation of maritime prohibition policies.
4	Qing Dynasty	During the early Qing period under Emperor Shunzhi, foreign trade policies were not entirely closed. Zheng Chenggong (Koxinga), operating under official permits, dispatched merchant vessels to Japan and Southeast Asia to procure urgently needed materials such as copper ingots.
5		In the Qing dynasty, wealthy merchants from Chenghai County in Chaozhou Prefecture capitalized on the sugarcane harvest season by acquiring large volumes of raw sugar, which were subsequently shipped via maritime routes to commercial centers such as Suzhou and Tianjin.
6		Additionally, brown and white sugar produced in Chaoyang County of the same prefecture were transported by merchant vessels to cities including Jiaxing, Songjiang, and Suzhou, where they were exchanged for cotton and textiles—thereby facilitating the integration of the local sugar economy into the broader economic network of the Jiangnan region.
7		During the Qing dynasty, shipbuilding technology had reached a high level of maturity. Most vessels had a carrying capacity of 300–400 tons and were extensively employed in both inland and maritime trade, thereby facilitating commodity circulation and enhancing regional economic connectivity.
8		According to Nanyue Youji by Chen Huiyan, Guangdong merchant vessels of the period transported both passengers and cargo. The local government regulated the passenger and cargo capacities, as well as fare structures, based on vessel size.
9		The Qing government closed the maritime customs offices in Jiangsu, Zhejiang, and Fujian, retaining only the Canton Maritime Customs (Yuehaiguan) as the exclusive port for foreign trade. It imposed strict controls on foreign commercial activity and limited the export volumes of strategic commodities such as silk and tea
10		Following the designation of Guangzhou as the sole legal foreign-trade port, Guangdong’s waterborne commerce entered an unprecedented period of prosperity.
11		Regulatory measures introduced by the Guangdong authorities stipulated vessel design parameters and tonnage limits, including prohibitions on tall masts. Passenger ferries were permitted to carry goods, provided freight charges were calculated according to the passenger fare system—demonstrating a combination of regulatory stringency and administrative flexibility in the management of water transportation.
12		To enhance oversight, the Qing government mandated that all civilian sea-going vessels be registered and assigned identification numbers visibly engraved on the bow or mast. Merchant vessels from different provinces were distinguished by specific paint colors applied to the hull and mast, facilitating inspection, licensing, and enforcement procedures.
13		The so-called “Red-Head Ships” (hongtou chuan), prominent in the Chaozhou-Shantou region, were long-distance trading vessels used by overseas Chinese merchants. These ships featured red-painted prows adorned with black circular motifs resembling pupils. This decorative convention, rooted in religious beliefs, was thought to provide spiritual protection and ensure navigational safety.

Maritime technological development during the Ming and Qing dynasties was characterized by multidimensional interaction and structural integration. From a technical perspective, military imperatives served as a primary driver. For example, the Ming-era Fu Ship combined hydrodynamic efficiency with defensive capacity through innovations including V-shaped hulls, watertight bulkheads, and multi-tiered combat platforms (events 1–2). By the Qing period, the introduction of codified ship registration systems (event 12) and standardized equipment protocols (event 11) reflected a shift from physical resistance to institutionalized defense strategies. With regard to policy regulation, the maritime prohibition (haijin) disrupted the technological lineage of the Zheng He fleet (event 3), whereas the establishment of the Canton Single-Port Trade System (event 9) and the state-merchant collaboration model (events 5–6) reconfigured the regional trade network. These reforms fostered the emergence of a new maritime system marked by formalized shipping routes (event 8) and centralized distribution hubs (event 10). Culturally, the watertight bulkhead technology in Fu Ships embodied kinship ethics (event 2), while the “red head and crow’s eye” motif on Red-Head Ships (event 13) blended vernacular belief systems with functional design. Together, these practices underscored the role of ship morphology as a material expression of political authority, regional identity, and religious symbolism.

## 5 Discussions

The analytical results of Section 3.2 are presented below. The central keyword clusters are anchored in ‘economy’ (Group A), ‘military’ (Group F), and ‘other’ (Group C), with ‘geography’ (Group D) forming a secondary cluster. Due to the smaller number of top 100 keywords in the ‘vehicle (Group B)’ and ‘politics (Group E)’ clusters, these clusters do not exhibit significant core content and are therefore considered ineffective clusters. This indicates that economic and military factors are the primary drivers, while geographic factors play a secondary yet supportive role. The term “trade” links the ‘economy’ (Group A) and ‘military’ (Group F) clusters, while co-occurring terms such as ‘resources,’ ‘haulage,’ and ship types like ‘Treasure Ship (宝船),’ ‘Turreted Junk (楼船),’ and ‘Cabin’ further substantiate their semantic interrelation. This suggests that the evolution of merchant ship design was primarily driven by the adaptive demands of trade-oriented functions. Trade-related events in the ‘Guangzhou’ region closely align with the ‘geography’ (Group D) and ‘military’ (Group F) clusters, underscoring the area’s geopolitical centrality in global maritime networks. The term ‘Ship’ serves as a link between the ‘Military (Group F)’ and ‘Politics (Group E)’ clusters. The co-occurrence of ‘Cargo Ship’ and ‘Merchant Ship’ with ‘government,’ ‘authorities,’ and ‘viceroy (总督)’ suggests that merchant vessels functioned not only as economic instruments but also as tools of political and military governance. This highlights the strategic role of merchant ships in state policy implementation, maritime security, and the projection of overseas influence. Place names such as ‘Guangzhou,’ ‘Fuzhou,’ and ‘Quanzhou’ appear frequently across both texts. Adjacent regions including ‘Guangzhou,’ ‘Foshan,’ and ‘Chaozhou’ demonstrate high Pearson RSD values and form multiple composite spatial clusters, indicating strong regional integration. This underscores the dual influence of locational geography (e.g., port accessibility and inland water routes) and shipbuilding technologies in shaping maritime development and regional geopolitical centrality.

The results of the analysis for section 3.3 are shown below. High-frequency terms such as ‘boat owners,’ ‘salt,’ and ‘merchant ship’ are closely associated with the ‘Qing Dynasty,’ forming a central cluster. This indicates that salt was an important export commodity during the Qing Dynasty and likely influenced the storage function of cargo ships. The terms ‘technology’ and ‘shipbuilding’ are closely linked with the Han and Sui Dynasties, forming a central cluster, highlighting the foundational impact of early Chinese shipbuilding technology on later merchant ship designs, which in the Ming and Qing Dynasties focused on trade and transport functions. During the Ming Dynasty, maritime trade in the Guangdong region was most active with India (TF = 26), followed by Sri Lanka (TF = 10), Japan (TF = 8), Arabia (TF = 7), and Korea (TF = 5). However, in the Qing Dynasty, apart from Japan (TF = 8), the activity of other countries mentioned earlier significantly declined, which may relate to changes in the trade system. In the Qing Dynasty, Guangdong’s shipping routes extended beyond neighboring seas to European regions, including Britain (TF = 11) and the Netherlands (TF = 6).

Section 4 analysis presents the following findings. Merchant ships of the Ming Dynasty were constructed primarily from teak and cedarwood, painted in red and yellow hues, and characterized by spacious, flat-bottomed hulls with streamlined, upturned bows. Teak refers specifically to Tectona grandis, a tropical hardwood native to South and Southeast Asia, which was widely known in maritime trade and utilized in Chinese shipbuilding during the Ming Dynasty. Qing Dynasty merchant ships incorporated lightweight and durable iron-nickel alloy, with hulls painted in more subdued colors (mainly blue-gray). These ships featured double-layered planks to enhance wind and wave resistance, and their bows were designed with a sharp taper. The Sha ship, designed for shallow-water navigation, emerged during the Qing Dynasty alongside the widely used Guang and Fu ships of the Ming era, forming the three primary ship types for Guangdong’s shipping network. From the Ming to the Qing Dynasties, the hull designs of Guangdong merchant ships evolved progressively from “wide and bulky” to “streamlined and agile” to “stable and durable.” The structural, technological, and decorative features of Guangdong vessels during the Ming and Qing dynasties exhibit a high degree of systematization, distinguishing them markedly from contemporaneous ships of neighboring countries and the West. These characteristics reflect not only the unique configuration of China’s state organization and technological systems but also the enduring continuity of its maritime cultural traditions.

The cultural morphology of ships is also manifested in their structural technologies and ornamental practices. As Huajie et al. [31] observed, significant divergences exist between Chinese and European vessels in symbolic systems and aesthetic perceptions: Chinese ships employed stylized motifs such as tiger heads and dragon eyes, coupled with high-saturation color palettes, to convey ritualistic meanings and protective symbolism. In contrast, European vessels utilized three-dimensional relief carvings to depict deities and heraldic emblems, thereby constructing a visual order grounded in religious and secular authority. Lee [32], in a comparative study of Chinese and Korean traditional ships, noted that Korean vessels predominantly adopted flat-bottomed, transverse beam structures suitable for coastal navigation, whereas Chinese ships typically featured V-shaped keels with transverse bulkheads, enhancing their ocean-going capabilities and compartmentalization. Hong [33] further expanded this comparison to include Japanese ships, highlighting that Chinese vessels such as the Sinan wreck employed bulkhead compartmentalization and mortise–tenon with iron-nail jointing techniques to maximize watertight integrity and offshore resilience. In contrast, Japanese vessels often used rope-bound keel–rib frameworks, which, although well-suited for littoral operations, were relatively deficient in waterproofing. These structural divergences underscore fundamental differences among China, Korea, and Japan in maritime strategies, technological traditions, and adaptive approaches to oceanic environments.

The historically embedded interplay of geopolitical structures and intercultural exchanges was pivotal in shaping the morphological characteristics of Guangdong merchant ships. During the Ming and Qing dynasties, the South China Sea and the East Asian littoral constituted a multilayered, interlocking maritime political geography. As a strategic port and institutionalized gateway for foreign trade, Guangdong’s vessels served not only commercial and military purposes but also embodied national sovereignty and cultural identity. The structural and decorative features of these vessels further reflect the complex influence of cross-cultural interactions. As demonstrated by Sasaki [34], transregional exchange and technological diffusion within the East Asian maritime trade network enabled the Guangdong shipbuilding tradition to incorporate Southeast Asian and Western innovations while retaining its indigenous craftsmanship. This process of “peripheral internalization” fostered a high degree of hybridity, adaptability, and strategic flexibility in the morphology of Guangdong merchant ships.

The evolution of Guangdong merchant ship design during the Ming and Qing periods was shaped by two key mechanisms: maritime insecurity and institutional trade regulation. Referencing the works from Ye [35] and Zeng [36], Table 10 lists historical events in which military threats and trade policies influenced ship hull structures. Rising piracy in the South China Sea prompted merchants to reinforce hull structures with thicker planking and elevated prows for defensive purposes [37]. By the late Qing, some vessels were even equipped with cannons, reflecting a growing need for self-protection in hostile waters [38]. Simultaneously, the implementation of the Guangzhou System centralized trade through a single port and expanded long-distance commerce with Europe [39]. This policy shift demanded ships capable of enduring extended voyages and carrying larger cargo loads, driving adaptations toward more streamlined, deep-draft hulls [40]. Thus, military threats and trade policies influenced ship morphology not through direct design mandates, but by reshaping risk profiles and logistical demands. These changes illustrate how geopolitical forces operated as structural drivers of technological adaptation in early modern maritime China.

**Table 10:** The influence of military threats and trade policies on the structure of Guangdong merchant ships.

No.	The influencing events of military threat
1	In the 24th year of the Jiajing reign, Qi Jiguang requisitioned 110 Hengjiang vessels in a single campaign and incorporated them into the naval fleet. Large civilian merchant ships were refitted by the authorities with additional decks and gunports, thereby directly converted into warships. The structural design of these vessels shifted from commercial to military purposes.
2	n the first year of the Chenghua reign, Han Yong oversaw the construction of 500 warships. Thereafter, local garrisons began to build and repair vessels on their own. From this practice emerged specialized types of Guangdong vessels such as the “Hengjiang Dashao” and “Wucao.” Civilian craftsmen were also conscripted, tasked with reinforcing bulwarks and raising forecastles according to military requirements, thus transforming commercial ships into naval craft.
3	Following successive raids by Japanese pirates at the Pearl River estuary, the Ming court imposed strict restrictions in eastern Guangdong, ordering the destruction of large vessels and the prohibition of double-masted ships. Civilian ocean-going junks of 1,000 tons were thereby limited to less than 200 liao (approximately 60 tons). Shipowners consequently refashioned large vessels into lighter dual-purpose ships, such as “Kuaixie” and “Hengjiang Shao,” suitable for both trade and combat.
4	When pirate confederations in South China—most notably the Red Flag and Black Flag bands—controlled the Pearl River estuary, the Qing court enforced stringent bans on civilian vessels such as the “Great Eight-Oared” and “Large Crow-Tailed” types, while mandating the installation of cannon mounts. To ensure self-protection, merchants converted their trading ships into “gunboat-style” Guangdong vessels, generally reduced to 150–250 tons, equipped with light artillery for defensive purposes.
5	After 1799, seven major pirate alliances, led by the Red Flag, Black Flag, and Yellow Flag groups, dominated the seas off Guangdong. Their ships, the largest carrying 500–600 men and the smaller still accommodating 200–300, were outfitted with foreign-style artillery such as yi pao and folangji. These fleets plundered merchant vessels and besieged coastal batteries along the route from the Pearl River estuary to the Qiongzhou Strait, constituting a sustained maritime military threat for more than a decade.
	The influencing events of trading policy
1	From the early Ming to the Zhengde reign, the tribute ship and maritime trade system was enforced, with the state monopolizing overseas commerce and prohibiting the private construction of large vessels for foreign trade. As a result, the thousand-dan oceangoing merchant ships of the Song and Yuan dynasties disappeared, and shipyards turned to the production of smaller vessels such as river transport boats, salt carriers, and agricultural craft.
2	In the 22nd year of the Qianlong reign (1757), the “Canton System” restricted foreign trade to Guangzhou alone. The maritime customs in Fujian, Zhejiang, and Jiangsu were closed, and all Western merchant ships were permitted to anchor and trade only at Huangpu in Guangzhou. The Canton Customs Office subsequently imposed an additional “ship fee” and levied taxes according to the length of the vessel.
3	In the 14th year of the Jiaqing reign (1809), the Regulations on Trade between Civilians and Foreigners designated separate anchorages for naval and merchant vessels. Merchant ships were permitted to dock at the inner harbor of Huangpu, while warships were restricted to the outer anchorage at Humen. The regulation further reiterated that “merchant vessels shall not exceed one zhang and eight chi in beam.”
4	In the 5th year of the Tongzhi reign (1866), the Regulations for Chinese Merchants on the Acquisition and Construction of Steamships were promulgated. The Zongli Yamen allowed Chinese merchants to purchase or build steamships, but restricted their use to cargo carriage and prohibited the transport of foreign artillery. The Canton Customs additionally imposed a tax of “one qian per net ton” on steamships.
5	In the 1st year of the Longqing reign, the Passport System was introduced, permitting overseas voyages but restricting beam, tonnage, and armament. Consequently, private oceangoing vessels were forced to contract to “below one thousand dan and within two masts.” Hulls became shorter and broader, and hatchways were reduced in size, enabling compliance with regulations and the acquisition of legal passports.

This study finds that by the late Qing Dynasty (post-1840), Guangdong merchant ships had evolved into multifunctional transport vessels, often equipped with reinforced prows and mounted cannons. Historical archives from the Ming Dynasty and earlier periods frequently document incidents of piracy and armed conflict involving merchant ships [41], with ship designs incorporating defensive features [42]. However, the East India Affairs Report notes that European merchant ships were generally expensive warships, whereas Qing-era (1616–1757) sailing vessels were purely merchant ships with no armament [43]. Based on the maritime history of Zheng Chenggong and archaeological records such as the Batavia City Diary [44] and the Journal of Jalanjar [45], Wang [43] also inferred that Chinese merchant ships before 1650 lacked active defensive capabilities. Lightweight and durable sailing vessels could adjust sail angles to accommodate varying wind directions, thereby reducing wind resistance [46]. In contrast, during the 17th and 18th centuries, the Dutch East India Company frequently incorporated heavily armed escort ships into its merchant fleets, reflecting a more institutionalized maritime defense system than that of the Qing dynasty [47]. Archaeological studies of square sail rigging support Reid’s [48] argument that mitigating natural disaster risks was a key driver in 17th-century merchant ship construction, which aligns partially with this study’s findings. Keywords such as “quicksand,” “wind speed,” and “passage” reflect the environmental considerations embedded in the design of Guangdong merchant ships.

**Fig 10:**
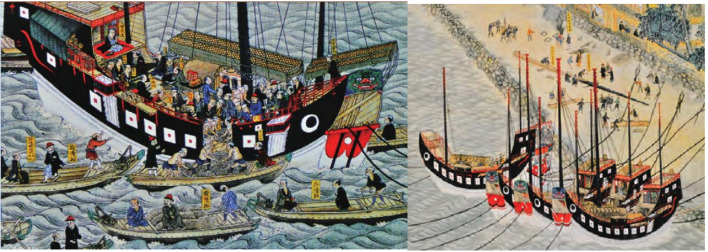
A large three-masted sailing ship of the Qing Dynasty en route to Nagasaki, Japan. a. A large three-masted sailing ship of the Qing Dynasty passing through Nagasaki, Japan. b. Manner of docking of ships after discharge of cargo.

A Japanese artist documented a Qing Dynasty merchant ship passing through Nagasaki, featuring bow and stern heights of 4.3 meters. This design likely balanced stability against large waves, while the bamboo mat sails, arranged in a three-masted configuration, facilitated long-distance voyages and large cargo capacities [49] (Fig 10). These three-masted ships could carry up to 500 tons of cargo [50], but Qing Dynasty trade regulations limited their loads to 40 tons and their crews to no more than 28 individuals [15]. Knowledge mining conducted in this study demonstrates a close relationship between the functionality of Qing Dynasty Guangdong merchant ships and their cargo capacities. The emergence of ship types such as “Salt Ship,” “Food Ship,” “Wood Ship,” and “Fishing Ship” [51] further supports this connection. In Fig 10a, a Japanese customs officer monitors the loading and unloading process at the ship’s stern. Japanese laborers employ traditional Chinese maritime unloading techniques, using ropes to secure and transfer goods onto transport vessels (Fig 10b). Merchant ships passing through Siam exhibited colors similar to those documented in Fig 10a for ships in Japanese waters. While the upper freeboard portions of these vessels were painted red and black, the remainder was primarily black. Guangdong ships were mandated to be painted red, while those from Fujian, Zhejiang, and Jiangsu were designated green, white, and black, respectively. The Sha ship depicted in Fig 10a resembles a fish, with eyes painted on the bow to symbolize the crew’s vigilance and determination to navigate correctly in the open sea [50].

**Fig 11:**
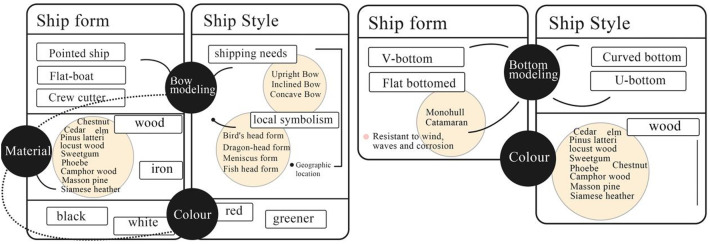
Morphological characterization of Guangdong merchant ships during the Ming and Qing dynasties.

This study delineates the evolutionary trajectories of Guangdong merchant ships during the Ming and Qing dynasties across three dimensions: bow and hull morphology, construction materials, and coloration (Fig 11). Regarding ship form, pointed bows and flat-bottom designs were suited to shallow waters and inland navigation, whereas V-bottom and U-bottom structures were more appropriate for oceanic voyages, providing greater stability. In terms of materials, construction prioritized durability, corrosion resistance, and adaptability to challenging marine conditions. Cedar and pine, known for their lightweight and anti-corrosive properties, were widely used, while iron reinforcements were applied to critical components such as the bow and bottom to enhance structural strength and resistance to wind and waves. Concerning color, red and green schemes were employed to differentiate vessels by region, thereby facilitating maritime trade supervision and taxation under the Qing administration. Ship bow modeling (e.g., bird’s head form, dragon-based form, fish head form) and bottom molding (e.g., monohull and catamaran forms) reflected a dual consideration for navigation functionality and cultural symbolism. The transition from monohull to catamaran forms highlights increasing demands for navigational stability and wave resistance.

The findings partially support the original hypothesis that a combination of geopolitical and economic interests drove the evolution of Guangdong merchant ships. Although the study draws extensively on historical sources to clarify the influence of geopolitical factors on the morphological evolution of Guangdong merchant ships, its empirical foundation remains limited, particularly with regard to the defensive functions of vessels in the mid-Qing period (1757–1840). Current discussions rely predominantly on textual evidence and speculative interpretations, without sufficient archaeological or technical data to capture the specificities and contextual uniqueness of these design transformations. Consequently, the functional attributes of merchant ships during this period risk being overgeneralized. Furthermore, the analysis of how geopolitical dynamics and technological advancements jointly shaped ship design primarily centers on mainstream trade routes. It neglects the technical adaptations and morphological evolution of vessels operating along atypical or secondary maritime networks. This omission constrains the generalizability of the findings and fails to capture the full complexity and heterogeneity of geopolitical–technological interactions.

At the morphological analysis level, the study’s treatment of ship dimensionality remains relatively coarse. It lacks a quantitative typological analysis of precise structural indicators such as hull length, angular curvature, and vertical height—benchmarks established in recent scholarship [52,53]. This limitation weakens the granularity and scientific rigor of the interpretation, thereby reducing the explanatory power of the study at the micro-structural level. For causal analysis of event relationships, it is recommended to adopt the hierarchical event coding framework proposed by Li et al. [54], which facilitates the construction of relational models, developmental trajectory models, and impact-layer models to elucidate the intrinsic logic between specific historical events and the morphological evolution of ships. To address these constraints, future research should integrate archaeological datasets, broaden the scope to encompass multi-scalar trade networks, and adopt refined morphological typology methods. These steps would significantly enhance the analytical capacity to explain the complex interrelations among geopolitical change, technological evolution, and material form.

## 6 Conclusions

A strong correlation is observed between the functional attributes of merchant vessels and the technologies of cargo transport. During the Ming and Qing dynasties, ship design prioritized structural stability and navigational performance, while progressively emphasizing multifunctionality and high load capacity to meet the growing demands of maritime trade. From the Kangxi to Qianlong reigns (1662–1796), various operational models emerged, including state-operated, privately managed, state–merchant joint ventures, and government-supervised commercial enterprises. These modes reflected the complex interplay between state regulatory authority and private commercial forces, underscoring the state’s dual role in oversight and facilitation of maritime commerce. Institutional reforms—such as the adoption of the “Canton System,” the establishment of additional customs offices, the regulation of taxation, and the creation of maritime trade supervisory bureaus—demonstrated the formalization of trade mechanisms and constituted strategic responses to threats like Wokou piracy. Geographic and climatic conditions further influenced navigators’ choices of vessel type, indicating a spatially embedded logic of adaptation. Key maritime nodes in Guangdong, including Guangzhou, Foshan, Chaozhou, and Macau, formed an extensive shipping network that extended inland via river systems into the South China Sea. By the late imperial period, Guangdong’s maritime routes reached distant regions such as India, Japan, Southeast Asia, and Great Britain, forming robust and diversified trade relations. The term frequency analysis of the TAG dataset underscores the decisive role of technological advancement in Guangdong’s shipping and shipbuilding sectors, alongside the direct influence of state policies and vessel development on maritime activities. The relatively high frequency of terms related to shipping vehicles—second only to economic terms—underscores the pivotal role of vessel functionality in facilitating trade.

To comprehensively investigate the factors driving the evolution of Guangdong merchant ships, it is necessary to analyze their period-specific characteristics and to consider the cultural relationships that shaped their morphological development. The evolution of Guangdong merchant ships during the Ming and Qing dynasties was significantly shaped by multi-scalar geopolitical dynamics. This process was shaped not only by external military threats and trade demands but also by internal trade policies and changes in social structures. The evolution of ship designs reflects the interplay between geopolitical environments and trade requirements.

However, this study is constrained by the lack of quantitative typological analysis of ship morphology. Future research may incorporate three-dimensional ship reconstructions and apply fractal dimension analysis to standardized planar grid projections of hull components to reveal underlying patterns of technological path dependency and functional evolution. Comparative analyses of design strategies and dimensional configurations across regional merchant ship types may further elucidate how geographic conditions, institutional environments, and cultural traditions are embedded in hull morphology. A multidimensional comparison between southern Chinese vessels and those from Southeast Asia, Japan, and the Korean Peninsula would also contribute to a deeper understanding of the structural diversity within East Asian maritime technological systems and the historical–institutional mechanisms shaping them.

## Supporting information

S1 Appendix A (Chinese Historical Terms): This document provides explanations of specialized terminology and proper nouns specific to the context of China during the Qing Dynasty, including tariff policies and introductions to relevant government officials. (XLSX)

S2 Appendix B (Text Material): This document records the raw data for text mining and details of the encoding. (XLSX)

S3 Appendix C (Ship Information): This document provides historical information and a functional overview of Qing Dynasty vessels. (XLSX)

S4 Appendix D (Ship Sample Data): This document compiles the ship type catalog for this research and classifies them. (XLSX)

S5 Appendix E (Word Frequency Statistics): This document presents the word frequency statistics results of text mining. (XLSX)
